# JhI-21 plays a role in *Drosophila* insulin-like peptide release from larval IPCs via leucine transport

**DOI:** 10.1038/s41598-018-20394-1

**Published:** 2018-01-30

**Authors:** Anna B. Ziegler, Gérard Manière, Yael Grosjean

**Affiliations:** 1 Centre des Sciences du Goût et de l′Alimentation, AgroSup Dijon, CNRS, INRA, Univ. Bourgogne Franche-Comté, F-21000 Dijon, France; 2 Present Address: Dendrite Differentiation Group, German Center for Neurodegenerative Diseases (DZNE), 53127 Bonn, Germany

## Abstract

Insulin is present all across the animal kingdom. Its proper release after feeding is of extraordinary importance for nutrient uptake, regulation of metabolism, and growth. We used *Drosophila melanogaster* to shed light on the processes linking dietary leucine intake to insulin secretion. The *Drosophila* genome encodes 8 insulin-like peptides (“Dilps”). Of these, Dilp2 is secreted after the ingestion of a leucine-containing diet. We previously demonstrated that Minidiscs, related to mammalian system-L transporters, acts as a leucine sensor within the Dilp2-secreting insulin-producing cells (“IPCs”) of the brain. Here, we show that a second leucine transporter, JhI-21, of the same family is additionally necessary for proper leucine sensing in the IPCs. Using calcium imaging and *ex-vivo* cultured brains we show that knockdown of *JhI-21* in IPCs causes malfunction of these cells: they are no longer able to sense dietary leucine or to release Dilp2 in a leucine dependent manner. *JhI-21* knockdown in IPCs further causes systemic metabolic defects including defective sugar uptake and altered growth. Finally, we showed that JhI-21 and Minidiscs have no cumulative effect on Dilp2 release. Since system-L transporters are expressed by mammalian β-cells our results could help to better understand the role of these proteins in insulin signaling.

## Introduction

Insulin is a major regulator of metabolism, growth, and development and is conserved in all metazoans. In mammals it is synthesized by the β-cells of the pancreas and is released into the blood in response to a meal to regulate the cellular uptake of nutrients such as carbohydrates. Previous work shows that branched-chain amino acids (BCAA) - especially leucine (Leu) - have profound effects on insulin secretion, directly stimulating insulin secretion from β-cells^1^. Leucine is also capable of activating the mTOR signaling pathway in insulin-responsive cells to stimulate protein biosynthesis in both dependent and independent manners^2–4^. Since ingestion of free leucine is discussed as an alternative therapy for Type II diabetes, further studies are clearly necessary to clarify the role of leucine in the insulin-signaling pathway^5–7^.

The fruit fly *Drosophila melanogaster* provides an excellent toolbox for genetic manipulations to decipher molecular and cellular pathways and has been extraordinarily useful in investigating questions that cannot be easily addressed in mammals. In *Drosophila*, eight insulin-like peptides (Dilp1–8) have been identified^8,9^. These hormones govern metabolism, growth, development, longevity, and certain behaviors^10–14^. Dilp1, -2, -3, and -5 are produced in and secreted by specific neurons within the brain called insulin-producing cells (IPCs)^8,15,16^. Dilp2 appears to be the most abundant Dilp in the IPCs, and it is secreted after the ingestion of proteins or only leucine^8,17,18^. Dilp2 loss-of-function mutants show diabetes-like phenotypes including decreased body weight and increased hemolymph carbohydrate levels^19^.

IPC neuronal activity leading to the secretion of Dilp2 is stimulated by leucine via indirect and direct pathways^17,18^. The slower indirect pathway involves the fat body, which is the fly equivalent of the mammalian liver and white adipose tissue. It has been shown that dietary leucine activates the TOR pathway in the fat body, which in turn releases hormonal signals to stimulate Dilp2 secretion from the IPCs^17^. We recently highlighted a second, faster pathway in which leucine directly acts on IPC activity^18,20^. Interestingly, this direct pathway is also present in mammals^18,20^. In *Drosophila* the direct sensing of leucine involves an L-type amino- acid transporter (LAT) called Minidiscs (Mnd). We postulated that Mnd allows the import of leucine into the IPCs and thereby regulates Dilp2 release. RNAi targeted against *mnd* specifically in IPCs abolished their ability to respond to leucine and hence diminished the leucine-induced secretion of Dilp2. Consequently, animals in which *mnd* expression was down-regulated in IPCs showed defects in sugar metabolism and growth^18^.

Based on its ability to transport leucine and its membership in the LAT family, Mnd was classified as a system-L transporter^21^. System-L is often the major nutrient transport system for large, branched, aromatic, and neutral amino acids in cells^22,23^. Two system-L transporters, Mnd and Juvenile hormone inducible-21 (JhI-21) have been described in *Drosophila*^21^. The functional similarity between these transporters was underlined by a study that screened for genes whose expression is regulated by Juvenile hormone (JH)^24^. JH is a key regulator of fly development and couples developmental processes to animal nutritional status^25–27^. These findings suggest that the two transporters not only exhibit similar substrate specificity but might also be involved in the same nutritionally regulated developmental processes. In this study we shed light on the role of JhI-21 in the Dilp2 release pathway by regulating IPC leucine transport.

## Material and Methods

### Drosophila stocks and media

The following fly strains were used in this study (BL numbers refer to Bloomington Stock Center reference numbers): *Dilp2*-Gal4 (BL37516)^8,28^; *w*^1118^ ^29^; UAS-*mCD8GFP* (BL32219); UAS-*GCaMP3.0* (BL32116); UAS-*NaChBac* (BL9468); UAS-*JhI-21*^*dsRNA*^ (108509 KK, VDRC stock center, Vienna); UAS-*Mnd*^*dsRNA*^ (11027KK, VDRC stock center, Vienna). Transgenic fly lines were outcrossed to the isogenic *w*^1118^ strain for five generations. Animals were reared in a 12:12 h light-dark cycle on a standard medium (6.5% yeast powder, 6.5% corn meal, 1% agar, and 0.5% methyl 4-hydroxybenzoate, an antifungal agent) unless otherwise stated. Larvae were starved on starvation medium as previously reported (1% agar, 1% sucrose, 1XPBS)^17^. In starvation medium + L-leucine (Sigma Aldrich), the amino acid was added to a final concentration of 20 mM when the temperature of the boiled food decreased below 65 °C. The restrictive medium for body-weight measurements contained 0.051% inactivated yeast, 1.244% corn powder, 0.45% sucrose, 0.3% methyl 4-hydroxybenzoate and 1% agar^17^.

### Calcium imaging

Calcium imaging was performed as previously described^18^. Briefly, L1 larvae expressing GCamP3.0 in their IPCs were collected 24 h AEL and reared at 25 °C until they reached the feeding L3 stage (~96 h AEL). Starved animals were transferred to starvation medium for ~24 h. Filet preparations^30^ were made to expose the brain to an artificial hemolymph (HL6) lacking Ca^2+^ ^18,31^. All unwanted tissues, such as the digestive tract and the fat body, were removed, the preparation washed with HL6 medium without Ca^2+^, and covered with 250 µl HL6 + 0.5 mM Ca^2+^. The preparation was placed under a 25x water-immersion objective on a Leica DM6000B microscope. GCamP3.0 fluorescence was excited using a Lumencor diode light source at 482 nm ± 25. Emitted light was collected through a 505–530 nm band-pass filter. Images were collected every 250 ms using an Orca Flash 4.0 camera and processed using Leica MM AF 2.2.0. Each experiment consisted of 30 seconds of recording (“before application”) followed by 90 s of recording after the addition of 250 µl of either HL6 solution (control) or HL6 solution + 40 mM leu (for a final leu concentration on the preparation of 20 mM) (“after application”). Baseline F_o_ was calculated by measuring the mean fluorescence within the IPCs, minus the mean fluorescence of brain regions not expressing GCamP, within 10 frames before the application of either HL6 or HL6 + 40 mM Leu (F_0_). After the application of either HL6 or HL6 + 40 mM Leu changes in fluorescence were calculated for each frame, in the same manner (F_i_). Changes in fluorescence ΔF/F_0_ were calculated as (F_i_-F_0_)/F_0_ and given in %_._ The peak fluorescence represents the frame with the highest ΔF/F_0_ after the application of either HL6 or HL6 + 40 mM Leu.

### Immunostaining of cross-sectioned larval brains

For sections larvae were cut in half and fixed in 4% PFA in PBS (pH 7.4) for 3 h at 4 °C. Fixative was then replaced with 25% sucrose in *Drosophila* Ringer´s solution, and tissue incubated overnight at 4 °C. Brains were dissected and embedded in Tissue-Tek (Sakura Finetek), frozen in liquid nitrogen, and sectioned at 14 µm. Sections were washed 2 × 10 minutes with TBS (100 mM Tris/HCl pH 7.4, 1.5 M NaCl) + 0.01% Triton (TBST) and blocked with 1% normal goat serum for 30 min at RT. Primary antibodies (mouse anti-GFP 1:100, Sigma-Aldrich #G6539; anti-JhI-21 1:250^32^) were diluted in blocking solution and incubated with the samples overnight at 4 °C. Sections were washed 2 × 10 min with TBST and incubated with secondary antibodies (anti-mouse IgG-Alexa Fluor 488, anti-rabbit IgG-Alexa Fluor 594, Thermo Fischer Scientific, 1:400) diluted in blocking solution for 3 h at RT. Samples were washed 2 × 10 min at RT and mounted in Dako mounting medium. Fluorescence was observed using a Leica TCS SP2 confocal microscope.

### Dilp2 quantification within whole-mount larval brains

Larval brains were fixed in 4% PFA in /PBS for 45 min at room temperature (RT) and washed for 6 × 10 min in PBS with 0.3% Triton X-100 (PBST). They were blocked in PBST + 10% normal goat serum (NGS; Sigma #G9023) for 1 h at RT. The brains were incubated with rat anti-Dilp2 (1:800)^17^ in PBST + 5% NGS overnight at 4 °C. The tissues were incubated at RT for 30 min and washed 6 × 10 min in PBST. The samples were labeled with anti-rat IgG-Alexa Fluor 594 diluted 1:400 in PBST + 5% NGS for 3 h at RT and washed 6 × 10 min with PBS. The brains were mounted in Vectashield mounting medium (Vector Laboratories), and confocal stacks were taken with a 1 µm step size using a Leica TCS PS2 confocal microscope. Maximum projections of image stacks were created using Leica confocal software. The mean Dilp2 immunofluorescence within each cluster of brain IPCs was calculated using the FIJI package (2.0.0-rc-43/1.51p; NIH) software. The mean fluorescence within a region adjacent to the IPCs served as background and was subtracted from the mean Dilp2 fluorescence within the IPCs. Finally, a mean value representing each genotype/condition was calculated.

### *Ex-vivo* brain cultures

Larvae were reared on standard corn/yeast medium until they reach the feeding L3 stage (~96 h AEL). They were washed, transferred to starvation medium, and starved for 24 h. The larvae were harvested and sterilized in 70% ethanol for 30 sec. before brains were dissected in *Drosophila* Schneider’s medium (Pan, Biotech) under sterile conditions. Brains were cultured for 16 h at 25 °C in *Drosophila* Schneider´s medium, with or without 20 mM supplementation with 20 mM leucine, after which they were fixed using 4% PFA. Dilp2 levels were determined by anti-Dilp2 staining and confocal microscopy as described above.

### Measurements of circulating levels of sugars

First instar larvae were collected 24 h after egg laying (AEL) and allowed to grow on standard medium. Feeding third-instar larvae (~96 h AEL) were starved for 24 h on starvation medium and then transferred to either starvation medium or starvation medium supplemented with 0,2% leucine. Hemolymph (2 µl) was collected from 8–10 larvae by using capillaries (Ringcaps, Hirschmann, 9600105). The hemolymph was diluted 1:10 in trehalase buffer (137 mM NaCl, 2.7 mM KCl, 5 mM Tris-HCl [pH 6.6]) and heated for 5 min at 70 °C. Hemolymph trehalose was converted to glucose by using 1 unit of porcine trehalase (Sigma #T8778) at 37 °C for 24 h^33^. The total concentration of glucose was measured using the colorimetric Glucose Hexokinase Assay kit (Sigma Aldrich, GAHK20) and evaluated using a SPECTROSTAR plate reader (BMG LABTECH) at 450 nm.

### Quantitative RT-PCR

Larvae were reared until the feeding L3 stage, and the RNA from six batches of 80 brains per genotype was isolated using TRIzol (Invitrogen). Samples were incubated with RNase-free DNase (Life Technologies) for 1 h at 37 °C. 1 microgram of RNA per sample was reverse transcribed using the iSCRIPT cDNA Synthesis kit (Bio-Rad). Real-time PCR was performed using a standard protocol (Applied Biosystems, Roche). Primers: *dilp2* (atcccgtgattccacacaag and gcggttccgatatcgagtta); *JhI-21* (ttgtttaccacggcgaaatag and ctttgtgacggaggagctaca); control gene: *actin* (caaattcaaggcgtgaaaact and tccagtcattcctttcaaacc).

### Adult body-weight measurement

Flies were allowed to lay eggs on standard fly food for 4 h. First-instar larvae were collected 24 h AEL and reared at a density of 30 larvae/tube at 25 °C on a restricted diet. Body weight was determined by weighing individual males 1 h after eclosion using a high-precision balance (Satorium R160P-*F1).

### Statistical analysis

All data were transferred to Prism 5.0d (Graphpad) for statistical analysis and tested for normality using the D’Agostino and Pearson omnibus normality test. Two data sets, which passed the normality test, were analyzed using the unpaired-*t*-test. Data sets, which were not normally distributed, were analyzed with the Mann-Whitney test. Three data sets, which passed the normality test, were analyzed by 1-Way-ANOVA followed by the Bonferroni’s *post hoc* test. Three data sets, which did not pass the normality test were analyzed by the Kruskal-Wallis test followed by a Dunn’s *post hoc* test. Normally distributed data with two nominal variables were analyzed using the 2-Way-ANOVA followed by Bonferroni’s *post hoc* test.

## Results

### JhI-21 is expressed in the IPCs

**Figure 1:**
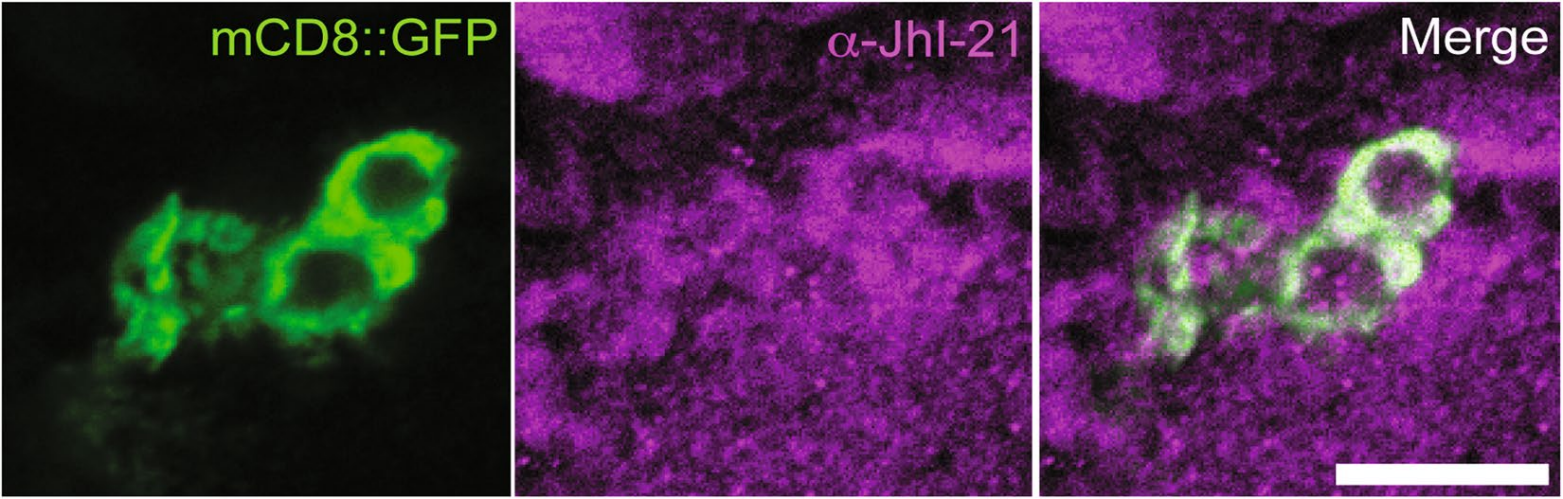
JhI-21 is expressed in the larval brain IPCs. Cross sectioned IPCs of feeding third-instar (L3) larvae expressing membrane-tethered GFP (mCD8::GFP, green) under control of *Dilp2*-Gal4 were co-stained with anti-JhI-21 antibody (magenta) show co-localization of GFP and JhI-21 within the cell membrane. Scale Bar = 10 µm.

We first investigated if JhI-21 is expressed in larval IPCs. Probing brains of feeding L3 animals with an anti-JhI-21 antibody revealed an overall broad expression pattern of JhI-21 in the central nervous system (Fig. 1, magenta). To identify the IPCs within this pattern, we used a *Dilp2-Gal4* transgene to express a membrane-tethered GFP (*UAS-mCD8::GFP*, Fig. 1, green). Within the JhI-21 staining pattern, we observed colocalization with this GFP signal, indicating that the IPCs are among the cells that express JhI-21.

### JhI-21 is involved in leucine sensing within the larval IPCs

When starved animals ingest food, their Dilp2-expressing neurons can directly sense the arrival of nutrients such as leucine. They respond to this change in nutritional status by increasing their activity, exhibiting a significant rise in cytosolic Ca^2+^-levels^18^.

**Figure 2:**
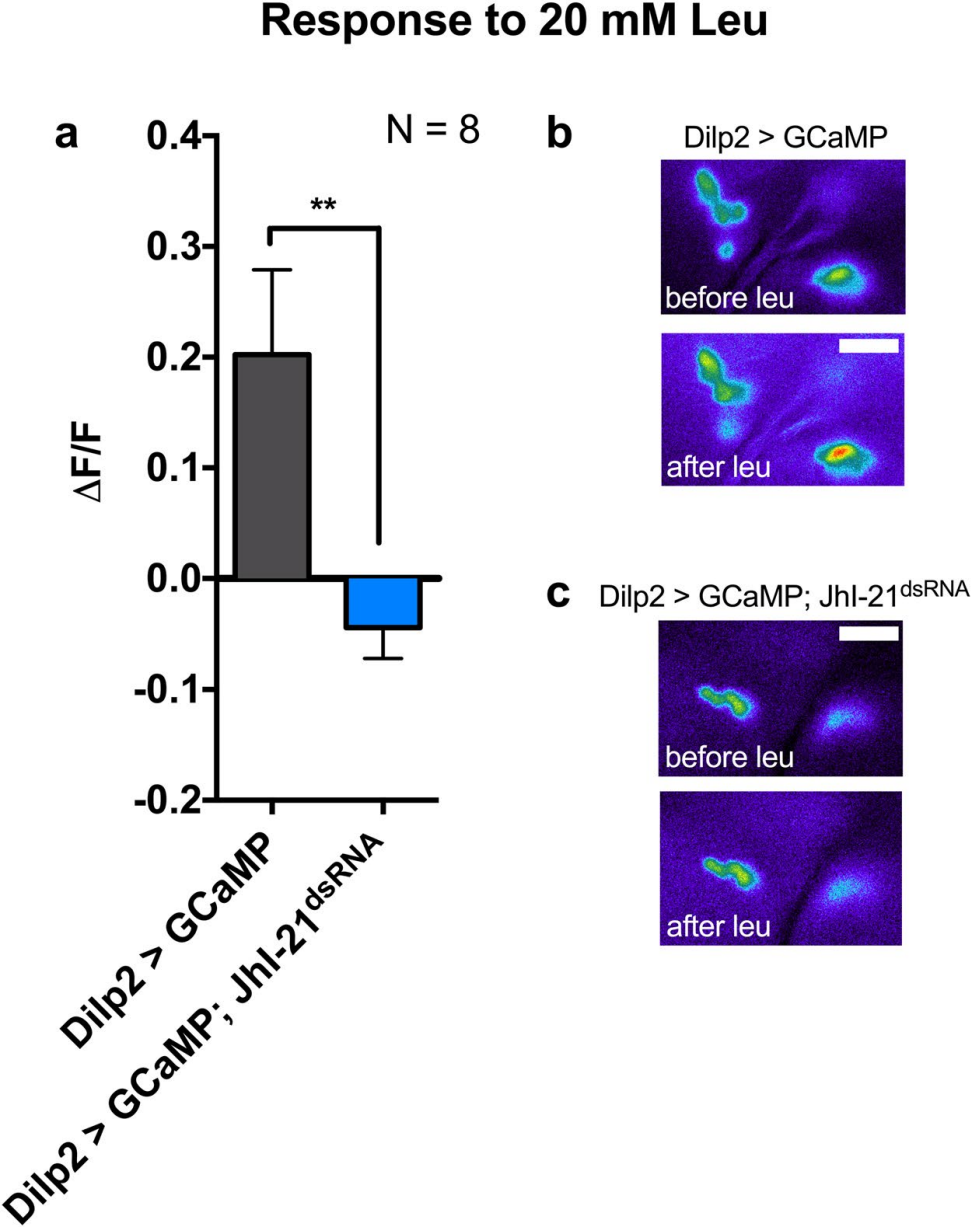
JhI-21 is involved in leucine sensing in Dilp2 expressing larval IPCs. GCaMP, a fluorescent Ca^2+^ indicator, was used to monitor neuronal activity of Dilp2-expressing IPCs of starved L3 larvae at feeding stage. It increases its fluorescence when Ca^2+^ concentration increases. (**a**) Probing larval brains with 20 mM leucine caused increased cytosolic Ca^2+^ levels in IPCs reflected by a rise in GCaMP fluorescence in the control genotype (*Dilp2* > *GCaMP*, grey bar). No increase in GCaMP fluorescence was observed when *JhI-21* expression was knocked down (*Dilp2* > *GCaMP; JhI-21*^*dsRNA*^, blue bar). Hence the neurons show no response to the increased leucine concentration surrounding the brain. (**b**) Representative pseudocolored images showing the increase of GCaMP fluorescence upon incubation of larval brains with 20 mM leucine. (**c**) Representative images showing that GCaMP fluorescence does not change upon incubation of the brains with 20 mM leucine when *JhI-21* is down-regulated in IPCs. Statistics in a: *t-*test, **p < 0.01; Data are mean ± SEM. Scale bar = 15 µm.

To test if JhI-21 is participating in this direct leucine sensing, we compared the rise in intracellular Ca^2+^-levels within IPCs in control animals and *JhI-21* knockdown animals using a Ca^2+^-imaging technique. Using *Dilp2-Gal4* to target the IPCs specifically, we expressed the fluorescent calcium sensor GCaMP3.0, with or without simultaneous knockdown of JhI-21 by *UAS-JhI-21*^*dsRNA*^. We then made filet preparations of starved feeding-stage third-instar larvae to expose their brain to an artificial hemolymph (HL6 medium) in which we could control the amino-acid composition. Unwanted tissues including the digestive tract or the fat body were removed to avoid crosstalk between tissues that are known to have an impact on Dilp2 secretion^17,34^. As previously reported^18^, 20 mM leucine increased the activity in Dilp2 > GCamP control IPCs (Fig. 2a, grey bar; Fig. 2b). This IPC response was abolished when JhI-21 was knocked down (Fig. 2a, blue bar; Fig. 2c).

**Figure 3:**
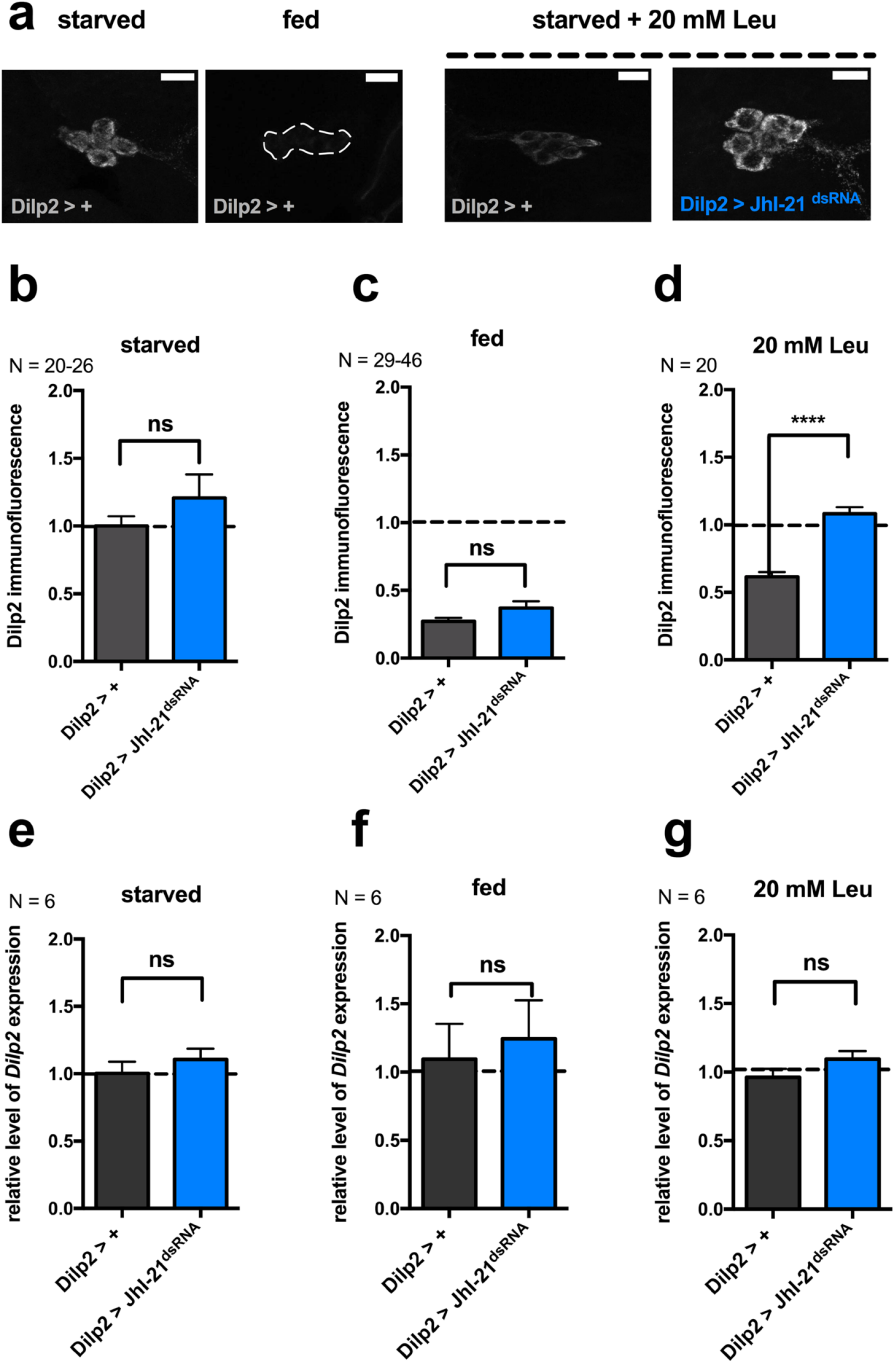
JhI-21 is involved in leucine-induced Dilp2 secretion. (**a**) Representative confocal stacks showing intracellular Dilp2 levels in a control genotype (*Dilp2*>+) or in a *JhI-21* knochdown (*Dilp2* > *JhI-21*^*dsRNA*^). Dilp2 levels were visualized by anti-Dilp2 immunolabeling. All larvae were raised on regular fly food until reaching the feeding L3 stage. They were then either starved for 24 h on starvation medium (starved), or continued to be fed with regular diet (fed), or starved for 24 h and fed with only 20 mM leucine added to the starvation medium (20 mM Leu). Finally, whole brains were dissected, probed with an anti-Dilp2 antibody, and observed under a confocal microscope. In the control genotype (*Dilp2-Gal4*>+) Dilp2 levels are high in IPCs of starved animals and low when fed on regular fly food. Dilp2 levels were also low when starved control animals were fed with starvation medium + 20 mM leucine. The same feeding condition did not cause diminished Dilp2 levels in the *JhI-21* mutant. (**b**–**d**) Quantified Dilp2 mean immunofluorescence intensities for control larvae (*Dilp2* >+, grey bars) and larvae expressing a UAS-*JhI-21*-RNAi transgene in their IPCs (*Dilp2* > *JhI-21*^*dsRNA*^, blue bars). (**e**–**g**) quantitative RT-PCR revealed equal *Dilp2* mRNA expression levels among genotypes and feeding conditions. Statistics in **b,c**: ns > 0.05, Mann-Whitney test; Statistics in **d**: ****p < 0.0001, *t-*test; Statistics in **e-g**: ns > 0.05, Mann-Whitney test. Data are mean ± SEM. Scale bar = 15 µm.

Previous work has shown that intracellular Dilp2 levels vary according to the feeding status of the animal. Dilp2 is stored within IPCs when the animals are starved and released upon feeding, as suggested by a decreased level of Dilp2 in IPCs^17,18^. Since knockdown of JhI-21 in IPCs abolished the normal leucine-induced activity increase, we wanted to investigate if Dilp2 secretion is also affected. For this purpose we compared the level of intracellular Dilp2 in IPCs from control larvae (*Dilp2*>+) to levels in *JhI-21* knockdown IPCs (*Dilp2* > *JhI-21*^*dsRNA*^) while varying the feeding conditions. Fed control larvae and *JhI-21* mutants showed lower intracellular Dilp2 stores than larvae that were kept on starvation medium lacking nutrients like leucine (Fig. 3a–c). The high Dilp2 level of starved animals was reduced when control larvae were fed on starvation medium supplied with 20 mM leucine, presumably indicating leucine-induced release (Fig. 3b vs. d, Dilp2>+, starved vs. 20 mM leu, *p* **** < 0.0001, Mann-Whitney test). By contrast, Dilp2 was not released when the experiment was performed using *JhI-21* knockdown larvae (Fig. 3d).

The increased amount of intracellular Dilp2 in *JhI-21* knockdown mutants could result from either impaired Dilp2 secretion or enhanced expression of Dilp2. We measured *Dilp2* mRNA levels in the previously described genotypes and feeding conditions to distinguish between these cases. No variations in *Dilp2* expression level due to the genotype or due to the feeding status could be observed (Fig. 3e–g). We conclude that the *JhI-21* knockdown in the IPCs causes an inability to secret Dilp2.

**Figure 4:**
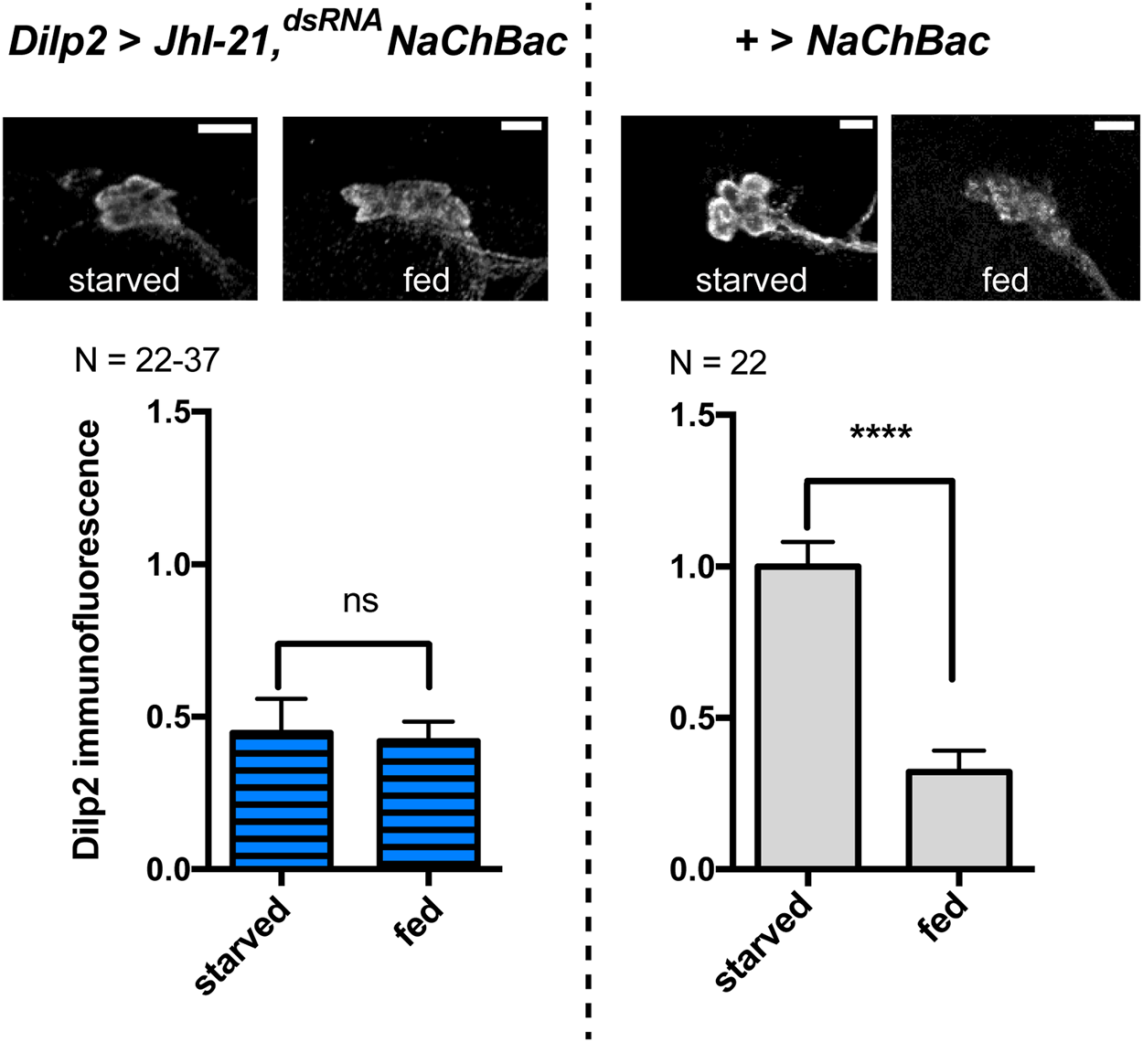
JhI-21 knockdown inhibits leucine sensing but has no impact on general IPC general function. L3 larvae were reared on standard medium until reaching the feeding L3 stage. Intracellular IPC Dilp2 stores were measured by Dilp2 immunolabeling in animals that were either starved or fed prior to brain dissection and Dilp2 labeling. Dilp2 is stored in IPCs of the control genotype (+>NaChBac) when the animals are starved. The low immunofluorescence level in fed animals reflects Dilp2 secretion upon feeding (right panels). Larvae that coexpress UAS-*NaChBac* and the *JhI-21* knockdown constructs in the IPCs have low stores of Dilp2 even if they have been starved prior to the experiment (left panels). Statistics: ****p < 0.0001, ns > 0.05, Mann-Whitney test. Data are mean ± SEM.

JhI-21 could affect Dilp2 release either by acting specifically on leucine sensing or by impacting general neuronal functions such as the ability to depolarize or to release vesicles. We used NaChBac, a bacterially derived sodium channel that has been shown to excite neurons in *Drosophila*, to test if JhI-21-depleted neurons are still excitable and competent to release Dilp2. IPCs that express NaChBac keep their original morphology indicating that NaChBac expression doesn’t kill the cells (Fig. 4, upper panel). We then compared intracellular Dilp2 levels in starved and fed animals, in which IPCs simultaneously express both NaChBac and the *JhI-21* knockdown construct. Dilp2 levels were dependent on the feeding status in animals that served as genetic controls (+>*NaChBac*, Fig. 4, right panel). When the IPCs were artificially excited by NaChBac, even starved animals had low intracellular Dilp2 levels (Fig. 4, left panel). This allows us to conclude that IPCs with reduced JhI-21 function exhibit specific defects in leucine-induced Dilp2 secretion, rather than a general functional impairment.

### JhI-21 is involved in cell-autonomous leucine sensing

**Figure 5:**
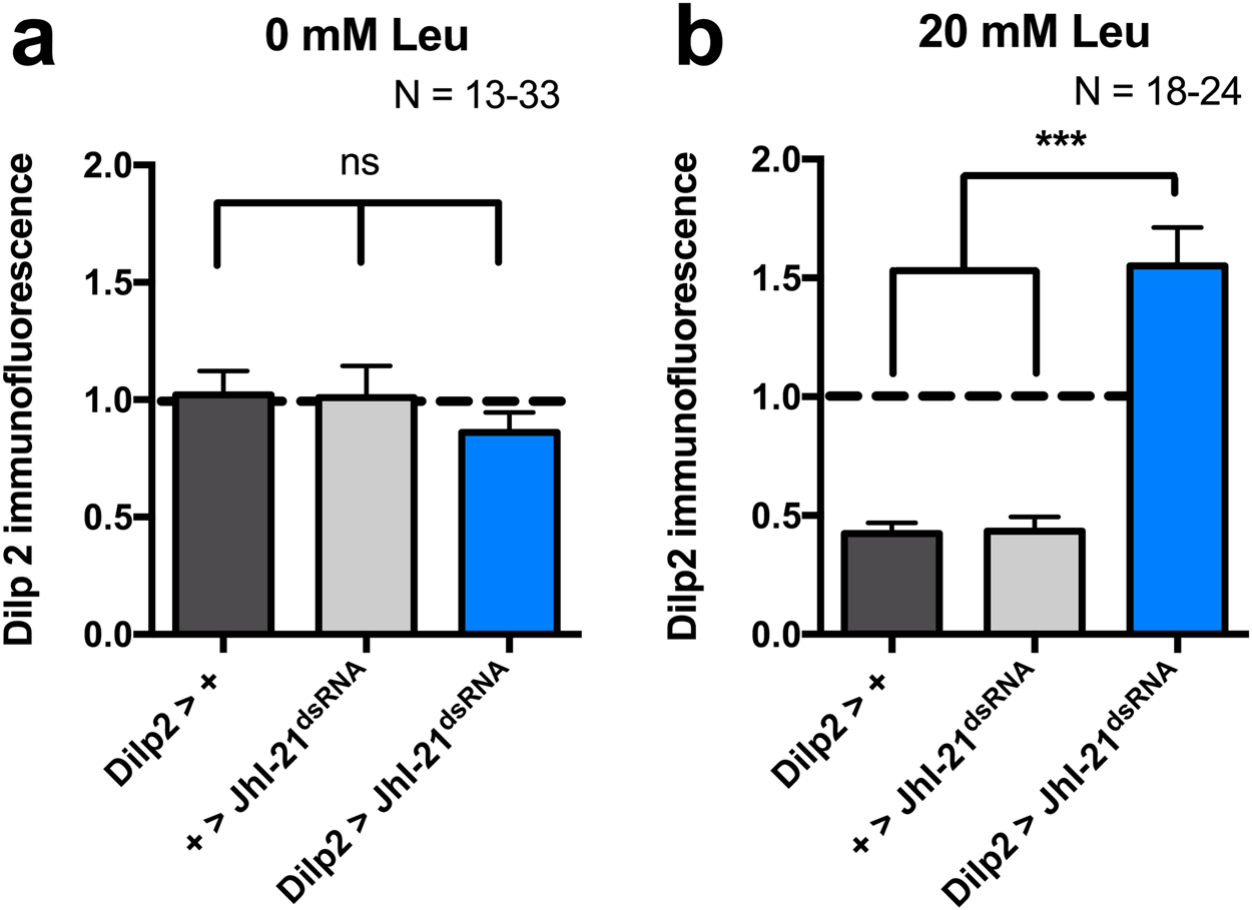
JhI-21 participates in autonomous leucine-sensing in IPCs. L3 larvae were reared on standard medium until reaching the feeding L3 stage and starved for 24 h. The brains of two control genotypes (*Dilp2*>+ and +>*JhI-21*^*dsRNA*^) and one *JhI-21* knockdown genotype (*Dilp2* > *JhI-21*^*dsRNA*^) were dissected and cultured overnight before fixation and Dilp2 immunolabeling. (**a**) Dilp2 immunolabeling was high in brains taken from starved larvae when cultured in regular *Drosophila* Schneider’s medium not supplied in leucine. (**b**) Dilp2 immunolabeling of control genotypes (grey bars) were low when brains were cultured in *Drosophila* Schneider’s medium that contained additional 20 mM of leucine. In IPCs in which *JhI-21* expression was knocked down (*Dilp2* > *JhI-21*^*dsRNA*^, blue bar), internal Dilp2 stores remained high. Statistics in a: ns > 0.05, 1-Way-ANOVA followed by Bonferroni’s *post hoc* test, Statistics in **b**: ***p < 0.001, Kruskall-Wallis test followed by Dunn’s *post hoc* test. Data are mean ± SEM.

Dilp2 release can be stimulated via two different pathways. The indirect pathway requires leucine sensing and activation of the TOR pathway in the fat body, which in turn releases hormonal signals that affect Dilp2 secretion from brain IPCs^17,34^. IPCs can also sense leucine directly via a mechanism that involves the LAT-transporter homologue Mnd^18^. To test if JhI-21 is similarly involved in the fat-body-independent direct leucine sensing by the IPCs, we investigated IPC Dilp2 levels in cultured control and *JhI-21-*knockdown brains. Larvae were raised on regular fly food until reaching the feeding L3 stage (~96 h AEL), and then starved for 24 h. We next isolated their brains, incubated them in Schneider´s medium for 16 h, and determined IPC Dilp2 levels by immunostaining. Brains of all genotypes assayed showed high levels of intracellular Dilp2 under these conditions (Fig. 5a). We next tested the ability of leucine to induce Dilp2 release by incubating the brains in *Drosophila* Schneider’s medium + 20 mM leucine. IPCs of control genotypes (either only the *Dilp2*-Gal4 or the *UAS-JhI-21*^*dsRNA*^ transgene alone) showed low Dilp2 levels under these conditions. In contrast, Dilp2 stores remained high when *JhI-21* was knocked down in IPCs (Fig. 5b). These results indicate that JhI-21 is involved in direct leucine-induced Dilp2 release from the IPCs, independently from the fat body.

### Metabolic consequences of JhI-21 knockdown in IPCs

**Figure 6:**
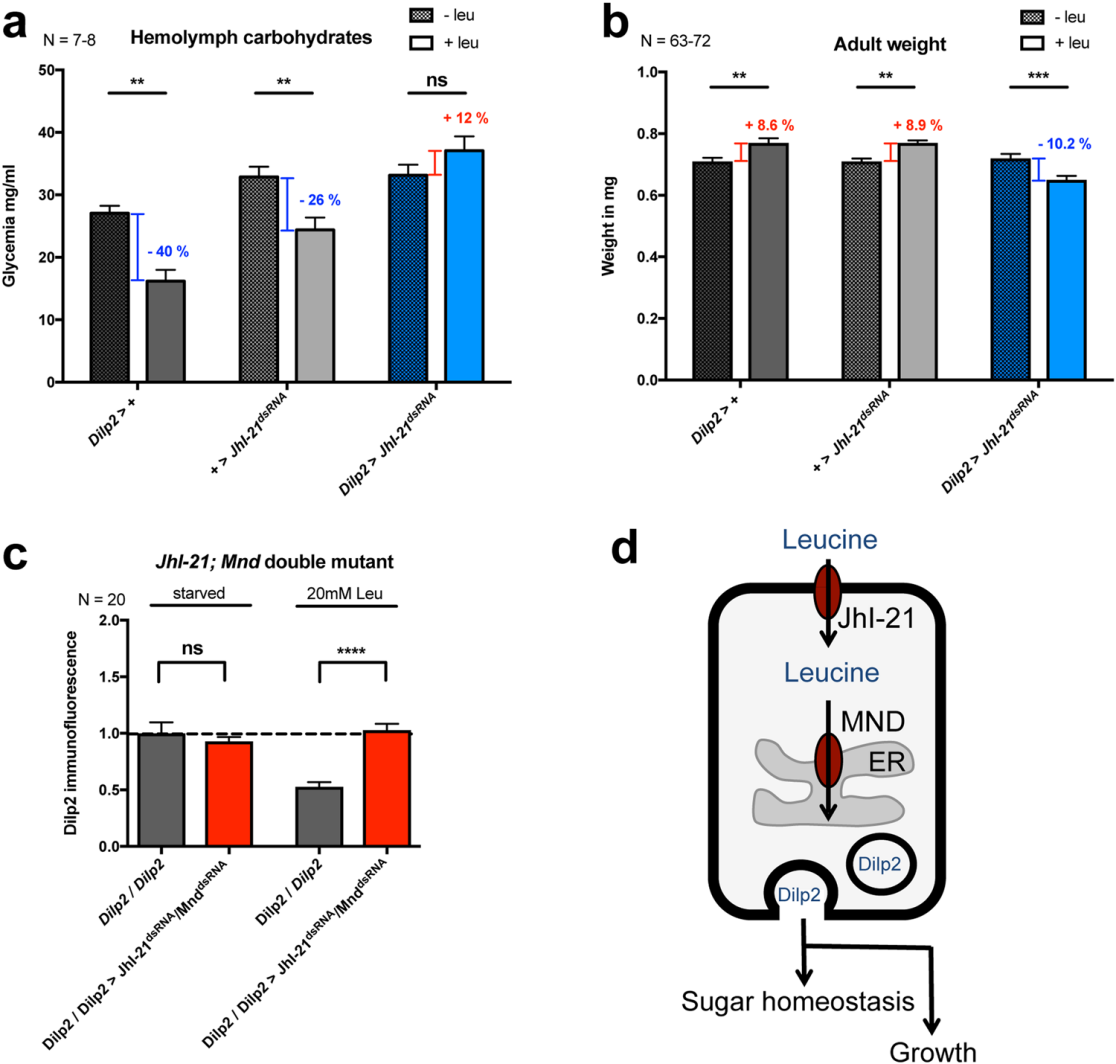
Carbohydrate levels and growth are affected by knockdown of JhI-21 in larval IPCs. (**a**) Carbohydrate levels (glucose + trehalose) were measured in hemolymph collected from larvae that were starved, or starved and refed only with leucine. Hemolymph carbodydrate levels decreased in both controls (*Dilp2*>+ and +>*JhI-21*^*dsRNA*^, gray bars) when larvae were fed with leucine. However, these levels did not change significantly when *JhI-21* expression in IPCs was down-regulated (*Dilp2* > *JhI-21*^*dsRNA*^, blue bars). (**b**) Weight of newly hatched adult males increased when control animals were fed with a restricted diet supplied with leucine. Knockdown of *JhI-21* in IPCs had the opposite effect on adult male weight (blue bars). (**c**) Larvae that coexpress *JhI-21* and the *Mnd* knockdown constructs (*Dilp2/Dilp2* > *JhI-21*^*dsRNA*^*/Mnd*^*dsRNA*^) in the IPCs did not show diminished IPC Dilp2 levels compared to controls when starved animals ingested 20 mM leu (**d**) Hypothetical model completing our first one^18^ for JhI-21 leucine sensing from IPCs to influence metabolism and growth in third-instar larvae. Our immunostaining experiments with various markers suggested that Mnd is localized mainly in the endoplasmic reticulum^18^, whereas JhI-21 is found at the cell surface of IPCs (Fig. 1). Statistics in **a** and **b**: ns > 0.05 **p < 0.01 ***p < 0.001, 2-Way-ANOVA followed by Bonferroni’s *post hoc* test, Statistics in **c**: ns > 0.05; p**** < 0.0001; Mann-Whitney test Data are mean ± SEM.

In *Drosophila*, Dilp2 secretion into the hemolymph affects general growth and carbohydrate homeostasis^15,28^. To measure if JhI-21 affects systemic Dilp2 signaling we first measured circulating carbohydrate levels in the hemolymph. Feeding third-instar larvae were starved or kept on a starvation medium that was supplemented with leucine. The presence of leucine led to significantly decreased hemolymph carbohydrate levels in the genetic controls (Fig. 6a, grey bars) but not in larvae with *JhI-21* deficient IPCs (blue bars in Fig. 6a). We next tested whether knocking down *JhI-21* in IPCs also affect growth. Animals were reared on either a restricted diet or a restricted diet supplied with leucine. Whereas leucine supplementation significantly promoted growth in the parental strains (Fig. 6b, grey bars) presumably reflecting increased Dilp2 secretion, *JhI-21* mutants showed no leucine-induced increase in body weight, but instead a significant decrease in their mass.

### JhI-21 and Mnd double RNAi Knockdown

Since JhI-21 and Mnd are both classified as LAT-1 like transporters^18,21,32^, and since both appear to play similar roles in IPCs and on metabolism (Figs 3 and 6,^18^), we wondered whether they could have cumulative or compensative effects on the Dilp2 signal detected in IPCs. To achieve this aim, third-instar larval brains of *Dilp2/Dilp2* > *JhI-21*^*dsRNA*^*/Mnd*^*dsRNA*^ genotype, raised in restricted feeding conditions with or without 20 mM leucine, were stained with an anti-Dilp2 antibody. This double RNAi avoided the release of Dilp2 compared to the control (Fig. 6c). This phenotype is similar to the single RNAi targeting either JhI-21 or Mnd (Fig. 3,^18^).

## Discussion

### JhI-21 is necessary for leucine sensing in IPCs

Dilp2 is released from brain IPCs in response to amino acids, particularly leucine^17,18^. An indirect fat-body mediated and a direct IPC-autonomous mechanism are involved in sensing the increase of leucine after feeding. The indirect mechanism involves the fat body. It senses the dietary amino acids like leucine. This mechanism also involves an amino acid transporter (slimfast) that is homologous to JhI-21^17,35^. Here, leucine-induced activation of the TOR-pathway within the fat body triggers the release of an unknown hormone, which in turn acts on brain IPCs to induce Dilp2 secretion^17^. We have recently discovered a second pathway, which involves a parallel, faster, direct leucine sensing within IPCs, requiring the Mnd leucine transporter^18,20^. We found that the IPCs respond to leucine by increasing neuronal activity followed by Dilp2 release. Compared to the other BCAAs leucine has the strongest effect on Dilp release from the IPCs^18^. This reaction occurs even when other tissues, such as the fat body, have been removed. Here we demonstrate that a second transporter of the same family, JhI-21, is also expressed by the IPCs and also regulates leucine-mediated activity and Dilp2 release. Knockdown of *JhI-21* abolishes the normal leucine-induced Dilp2 release. Larvae deficient in IPC *JhI-21* expression therefore suffer from metabolic derangements including increased hemolymph carbohydrate levels and growth defects (Fig. 6d).

In vertebrates, insulin signaling induces the uptake of glucose from the blood. Once absorbed this simple sugar can be stored, used as fuel, or metabolized into other compounds. Similar to insulin signaling in mammals Dilp2 secretion in *Drosophila* could activate in *Drosophila* downstream signaling pathways that might lead to the uptake of nutritionally delivered carbohydrates from the hemolymph into cells.

Our research therefore strongly supports a recent hypothesis in which LATs may have a dual transporter/receptor function. In this model they are not only passively transporting amino acids but also actively sense the size of the extra-/intracellular amino acid pool. They are therefore located upstream of intracellular pathways which might regulate anabolic or catabolic processes^36^.

### Functional relevance for JhI-21 in Dilp2 neurons

JhI-21 and Mnd have been previously characterized as leucine transporters and therefore defined as system L transporters^21^. Here, we show that JhI-21 is necessary for leucine sensing and proper Dilp2 release in IPCs. Although JhI-21 and Mnd are both classified as system-L transporters we could not observe a functional compensation when each transporters was knocked down separately. This could be explained by their differential sub-cellular expression since JhI-21 seems enriched at the plasma membrane (Fig. 1), and Mnd is mostly detected at the endoplasmic reticulum^18^. Indeed knockdown of the transporters separately or together blocks Dilp2 release from the IPCs in the same manner (Fig. 6c). This indicates that both may be essential actors within one pathway and have non-redundant functions within the direct leucine-sensing pathway in IPCs. This finding is in agreement with the previous characterization of Mnd and JhI-21 leucine transport capabilities in S2 cells^21^.

In mammals two transporters of the LAT family make up system-L transport: LAT1 (SLC7A5) and LAT2 (SLC7A8)^37^. These transporters are often co-expressed in cells and import neutral L-amino acids^38–41^. Nevertheless, they differ in their substrate specificity. While LAT2 transports many L-amino acids LAT1 has a rather narrow specificity transporting large L-amino acids^42^. However, their individual roles are still under investigation.

There are various plausible explanations for the frequently described co-expression of LAT-1 and LAT-2 transporters. First, they could be expressed in different compartments within the same cell or they might be polarized in different directions. Second, they may have different influx and efflux characteristics. Third, they might co-transport other substrates than leucine, which are essential for proper cell function.

On the other hand the function of LAT1 and LAT2 within the same cell has been investigated in several cell types^38–41^. One good approach that could help to understand the functional relevance of LAT1 and LAT2 co-expression was made in APRE-19 cells by Yamamoto and colleagues^38^. The authors suggest that both transporters might be localized on the plasma membrane but on opposite sites. They further report that LAT1 might predominantly import while LAT2 might rather export leucine^38^.

A similar scenario could be possible for JhI-21 and Mnd in *Drosophila* IPCs and could explain the necessity of co-expression of the two functionally related transporters. In this case one transporter could import leucine to induce Dilp2 secretion while the second transporter exports it, thereby terminating the signal. Further studies concerning the subcellular localization and influx/efflux affinities are clearly necessary to test this hypothesis.

JhI-21 or Mnd could also co-transport other amino acids than leucine, which might be equally necessary for proper amino-acid sensing. Since transporters of the LAT family are obligatory exchangers, they are in need of certain intracellular amino acids that are available for exchange with extracellular leucine. It is therefore possible that one transporter imports an amino acid that can be used for exchange against leucine by the second transporter. However, in mammals such interplay between two system-L transporters has not been proposed, rather only between LAT1/LAT2 and SLC7A9 family members^43^.

LAT-1 and LAT-2 in mice as well as JhI-21 and Mnd in Drosophila are not only expressed in Dilp secreting cells, but also in other cells^32,44^. This rather broad expression profile hypothesizes a diverse cellular role through leucine or other amino acid transport. For example, JhI-21 coevolves with glutamate receptors and impacts NMJ glutamatergic physiology to influence locomotor activity in Drosophila Larvae^32^. What might these transporters be doing in these none-insulin/Dilp secreting cells? Are they generic leucine transporters? What are their amino acid transport activities in these cells? Do they activate identical cellular and molecular pathways? These are fascinating questions, which still remain to be addressed.

### From *Drosophila* IPC to mammalian β-cell function

Pancreatic β-cells, which are the mammalian functional ortholog of *Drosophila* IPCs, release insulin when extracellular leucine levels are high^1^. However, the proteins that act as leucine sensors in those cells are still unknown. Here, we provide evidence for the involvement of system-L transporters in direct leucine sensing. Therefore it would be reasonable to investigate if this function has been conserved between insects and vertebrates during evolution. According to the β-cell gene atlas (www.t1dbase.org), LAT1 and LAT2 are also co-expressed in pancreatic β-cells in human, mouse, and rat. The expression level of both transporters is nearly equal in human and rat β-cells but not in mice, where LAT1 is much more highly expressed. Since JhI-21 and Mnd are functionally and phylogenetically related to LAT1 and LAT2, it would be interesting to knock down system-L transporters in pancreatic β-cells and to test whether they have an impact on leucine sensing and on insulin release. An alternative promising strategy would be also to try to rescue the *Drosophila* Dilp2 secretion phenotype with mammalian isoforms. This would help to break down the barriers related to human insulin diseases.
